# German Review

**Published:** 1891-08

**Authors:** Leonard Pearson


### Selections from Foreign Journals.

### *GERMAN.*

By Leonard Pearson, B. S., V. M. D.

### Inflammation of the Udder of a Cow.

Veterinarian Thum.—Thalmassing.

Thum was called to see a cow, which he found to have a swelling of the udder, that extended from the navel to the perineum. The swelling of the udder itself was hard, and sensitive to pressure; that part of the swelling in front and behind the udder was œdematous. The right front quarter was larger than the others, and a light brown fluid, containing cheesy fragments, could be drawn from it through its teat. This discharge did not have a bad odor. The other three teats yielded small quantities of seemingly normal milk.

The owner stated that the cow had given birth to two dead bull calves eight days before this visit, and that the enlargement of the udder had commenced a week before the delivery. With the exception of a little weakness in the hind quarters, the cow's general condition was good. The treatment prescribed consisted of steaming the udder, thorough milking, and the application of the following salve.

| | | |
|---|---|---|
| ℞ | Acid. Salicy., | 2.5 solut. |
| | Spirit., | q. s. |
| | Adip. Suill., | 50.0 m. fl. |
| ♏. | | |

At the next visit, six days later, it was found that the local condition was much worse and the cow would not eat. The udder was so large that it nearly touched the ground; the œdema in front and behind it had increased; the temperature was 40.5° C., and the pulse 120. A point of the right front quarter was found to fluctuate, and through an incision made there eleven litres of yellow fluid were drawn off. Exploration of the cavity revealed the presence of a great quantity of floating shreds of tissue, which could be easily detached and removed. It was found that the cavity of this cyst had no connection with the milk cistern, and hence did not communicate with the outer air.

To this fact was probably due the absence of the micro-organisms of putrefaction and, hence, of bad odor in the cystic contents. Since it was impossible to thoroughly cleanse the cavity of the partly detached tissue with the hand on account of the smallness of the opening, and the fact that dangerous hemorrhage followed an attempt to extend the incision an instrument of the shape of a small hoe, with a handle twenty inches long, was constructed for the purpose. The treatment consisted, now, in removal of the shreds, and injections of the cavity with two to three per cent. solution of creoline. Large masses were brought away each day, for some fourteen days; one day a piece that weighed one and one-half pounds was removed. The cavity was so deep that the twenty-inch-long instrument could be hidden in it. Good healthy granulation commenced a month after the first operation, and in three weeks more the cure was complete. At this time the cow gave nine litres of milk per day, from the three healthy quarters.

It is stated that the owner afterward reported that the cow had been chilled, and an inflammation of the entire udder followed, so that the animal had to be fattened for the butchers. *Wochenschrift für Thierheilkunde, No. 24, 1891.*

---

### Vomiting of Horses.

Army Veterinarian—Duvinage.

The view that horses only vomit when the stomach is ruptured is very generally accepted, but the author cites two cases to show that this is not the only cause. Paralysis of the cardiac orifice of the stomach is necessary to vomiting, but this can be caused by the presence of certain chemical substances, by over-filling the stomach or by distention with gas. In the first case cited, the horse showed severe colicky symptoms while at work. The evening before he had eaten his food as usual. Violent retching was followed by the discharge of large quantities of sour-smelling food through the nostrils. With the exception of slight depression the horse had fully recovered by evening. The horse had received hay and green food with his rations, and the supposition is, that the distention of the stomach by gas was the cause of the vomiting.

In the other case the horse vomited at intervals for six hours. The vomiting was accompanied by severe retching and, in the intervals, coughing and depression were marked. The following symptoms were noticed: the visible mucous membranes were dark red; temperature 39° C.; pulse weak, and seventy-two beats in the minute; artery hard, forty-eight respirations per minute; trembling and tottering; staring expression of the face and peristalsis weak. On the lower third of the neck was a swelling, the size of a fist. The animal died, and the autopsy revealed a mass of food obstructing the duodenum. The stomach was dilated to three times its normal size, by gas; its wall was throughout, intact. A rupture five inches long was found in the left pillar of the diaphragm. The plug in the duodenum was the size of a goose egg; and the stomach contained, besides the gas, a quantity of semi-fluid. Plugs of food were also found in front of the point at which the œsophagus passes through the diaphragm, and on the lower part of the neck.

Duvinage is of the opinion that the rupture in the diaphragm was due to the pressure exerted, through the stomach, by the abdominal muscles; and that the hemorrhage following the formation of this tear produced the death of the animal.

The writer draws from these cases the conclusion that rupture of the stomach is not necessary to vomiting in the horse. *Berliner Thierarz. Wochs. No. 17, 1891.*

---

### Treatment of Malignant Tumors.

Professor von Mosetig.

Prof. v. Mosetig gave his experience in treating malignant growths, with injections of various kinds, at a recent meeting of the Vienna Medical Society. He has experimented with drugs of all sorts, including lactic acid; but, until he tried aniline coloring matters, with unsatisfactory results; it occurred to him that since the cells making up the tumors have less vital resistance than those of sound tissue, a dye that would stain their nuclei would probably destroy them, and not injure those of healthy tissue. An experiment in which he made injections of a solution of arsenic-free, aniline dye into a sarcoma, gave a favorable result. Now that pyoctamcin has come into such prominence, Prof. von Mosetig has tried injections of one five-hundredth and one three-hundredth of this substance each second or third day, directly with the tumor. He has found that sarcomas treated in this way do not disintegrate but dry up and contract upon themselves. Prof. von Mosetig is still experimenting and hopes to find a still more rapidly-acting substance.

Prof. Billroth gave as his opinion, that malignant tumors could be destroyed in this way. *Allg. Wiener Med. Ztg., No. 5, 1891.*

---

### Prolapsus of the Uterus following Abortion.

Army Veterinarian Wassersleben.

A mare aborted an eight month's colt, and twenty-four hours later a prolapsus took place. A subcutaneous injection of morphine was at once made, the womb washed with a solution of creoline, and returned. The mare was now placed in a stall, in which her hind feet were considerably higher than the front, in order to stop the pressing, but without success, as the uterus was repeatedly forced out. The patient was then taken into the riding-ring and walked, while the arm of the operator was kept in the vagina, as a truss. The pressing soon stopped, so that the arm could be removed; but, in a quarter of an hour, commenced again so that the prolapsus again occurred. A dose of 30.0 gms. of chloral was then given and, as it had no effect in an half an hour another dose of the same size was given. This did not entirely stop the pressing, but checked it. The uterus now hung down fully twenty inches, was blue in spots and the lower one swollen to the size of a child's head; it was washed again with creoline solution and replaced. The mare was now walked for two hours and given no time to stop to press, and the prolapsus did not occur again. The subsequent treatment consisted in injections of one two-thousandth solution of corrosive sublimate, and in one week the mare was put to work. *Zeitsch für Vet. Kund., No. 2, 1891.*

---

### Facial Paralysis.

Army Veterinarian Reinke observed a case in which both facial nerves were paralyzed. This was shown by the flacid hanging of the lips, inability of the horse to move them, difficulty in the prehension of food and in chewing. The affection recovered under the subcutaneous injection at first of veratrin, and afterwards of strychnine (0.1 gram each third day).

---

### Sodium Chloride as a Therapeutic Agent.

The use of a solution of sodium chloride subcutaneously in the treatment of hernia was mentioned in the June number of this journal. It was recommended by Imminger, a German veterinarian. Luton and Lenormand in *La Presse Vet.* now commend it in umbilical hernia. Their method is to inject, with a hypodermic syringe, a small quantity of a saturated solution of common salt, under the skin on each side of the rupture. On the following day there is a marked odematous swelling which disappears gradually in some five days. At the end of this time the swelling is no larger than a dollar, and complete recovery soon results. *Berl. Thierârz. Wochs., No. 26, 1891.*

---

### The Italian Laws for the Sanitary Control of the Meat and Milk Supplies.

A law under this title was given out August 3, 1890, which might serve as a model for all regulations of this sort. The thoroughness of this law reflects great credit upon those who drew it up. All the precautions that modern investigation have shown to be desirable have been observed. In regard to the meat supply the provisions are for: 1st, The compulsory examination of all butcher animals; 2nd, The erection of public slaughter-houses in all cities of over six thousand inhabitants; 3rd, The conferring upon veterinarians the direction and supervision of these slaughter houses; 4th, The sale of poor quality, but not unhealthy meat, apart from the prime sorts; 6th, Strict rules for the inspection of imported meats.

The rules regulating the milk supply are fully as excellent and thorough. They provide for: 1st, The licensing of every dairy; The examination, by a veterinarian, of every cow before she is allowed to enter the dairy; 3rd, The compulsory reporting of each case of sickness among the cows of the dairies; 4th, The regulation of the sale of the milk from sick cows; 5th, The closing of dairies upon the breaking out of an epidemic. *Zeits. für Fleisch und Milch hygiene, No. 10, 1891.*

Laws of this sort are not peculiar to Italy, but fully as stringent regulations exist in France and Germany, besides many of the smaller countries of Europe. They are not made, as one writer has declared, to give the law makers and officers something to do, but because investigation has shown that many diseases are caused by the consumption of impure and diseased food of these classes, and because experience has shown the satisfactory effects of a well-regulated meat and milk inspection. Regulations of this class are, each year, becoming stricter in all parts of

Europe. In Saxony, for example, it is expected that laws will soon be adopted providing for the compulsory examination of every butcher animal killed, in town or in the country, by an expert. At the present time it is required that thirty-six microscopical examinations be examined for trichinea from every hog that is killed in the kingdom. England is the last of the European countries to take up this question, but from the energy with which it is now being discussed, there can remain but little doubt that she will fall in line and soon adopt similar laws. In this case the United States will be the last, and only one, of the highly civilized countries without a regulated meat inspection ; and this in the face of the fact that she is the greatest beef and pork producing country in the world! When the nations that should be our greatest buyers are so stringent in regard to their own home-raised products we can not be so imprudent as to ask that they accept foreign goods without a clean and authentic certificate of health. Meat inspection—careful, thorough meat inspection—must eventually come in the United States, and the sooner this time comes the better it will be for our foreign trade—for our shippers and producers.

